# Antibacterial Effects of Theaflavins against *Staphylococcus aureus* and *Salmonella paratyphi B*: Role of Environmental Factors and Food Matrices

**DOI:** 10.3390/foods12132615

**Published:** 2023-07-06

**Authors:** Jun Wang, Hongyan Shan, Ping Li, Yanan Liu, Xun Zhang, Jingguo Xu, Songnan Li

**Affiliations:** 1 School of Tourism and Cuisine, Yangzhou University, Yangzhou 225127, China; 007232@yzu.edu.cn (J.W.);; 2 Sericultural & Agri-Food Research Institute Guangdong Academy of Agricultural Sciences, Key Laboratory of Functional Foods, Ministry of Agriculture and Rural Affairs, Guangdong Key Laboratory of Agricultural Products Processing, Guangzhou 510610, China; 3 Joint International Research Laboratory of Agriculture and Agri-Product Safety of the Ministry of Education of China, Institutes of Agricultural Science and Technology Development, Yangzhou University, Yangzhou 225009, China

**Keywords:** theaflavins, diameter of inhibition zone, temperature, pH, NaCl, skimmed milk powder, lecithin, sucrose

## Abstract

This study aimed to investigate the effects of different environmental factors (temperature, pH, and NaCl) and food matrices (skimmed milk powder, lecithin, and sucrose) on the antibacterial activity of theaflavins (TFs) against *Staphylococcus aureus* (*S. aureus*) and *Salmonella paratyphi B* (*S. paratyphi B*). TFs showed a larger diameter of inhibition zone (DIZ, 12.58 ± 0.09 mm–16.36 ± 0.12 mm) value against *S. aureus* than that of *S. paratyphi B* (12.42 ± 0.43 mm–15.81 ± 0.24 mm) at the same concentration (2–10 mg/mL). When temperatures were 25–121 °C, the DIZ of TFs against both *S. aureus* and *S. paratyphi B* was not significantly different. As pH increased from 2 to 10, their DIZ values decreased significantly from 16.78 ± 0.23 mm to 13.43 ± 0.08 mm and 15.63 ± 0.42 mm to 12.18 ± 0.14 mm, respectively. Their DIZ values increased slightly as the NaCl concentration increased from 0.2 mol/L to 0.8 mol/L, while their DIZ values decreased significantly for skimmed milk powder concentrations in the range of 20–120 g/L. Regarding the concentrations of lecithin and sucrose were 2–12 g/L and 10–60 g/L, their DIZ values showed no significant change against *S. paratyphi B*, but an increased trend for *S. aureus*. Under the above different environmental factors and food matrices, TFs maintained excellent antibacterial activity against *S. aureus* and *S. paratyphi B*, providing a theoretical guidance for applying TFs as novel antibacterial additives in the food industry.

## 1. Introduction

Microbial contamination is a common food safety issue with potentially serious consequences, including food spoilage and foodborne illnesses, both of which pose significant risks to public health and result in substantial economic losses [1,2]. Therefore, inhibiting the microbial growth in the foods while maintaining quality, freshness, and safety, referred to as food preservation, becomes necessary and meaningful. The food industry has been using preservatives, such as nitrates, for many years to extend food shelf-life by disrupting the activities of pathogenic and spoilage microorganisms [3]. However, the above preservatives are synthetic substances that may cause problems for public health, such as allergic reactions [4]. Therefore, natural preservatives have received widespread attention in food preservation with ensured food quality, reduced health hazards, and improved antibacterial efficiency; and they have gained wide attention in food preservation due to their non-toxic, efficient, and operable characteristics [5,6].

Theaflavins (TFs) are antioxidant polyphenols with a reddish color formed by the condensation of flavan-3-ols in tea leaves during the fermentation of black tea, and their content accounts for 0.5–3.0% of the dry weight in black tea [7,8,9]. Theaflavin and its derivatives, including theaflavin-3-gallate (TF2A), theaflavin-3′-gallate (TF2B), and theaflavin-3,3′-digallate (TF3), contribute to the quality and bioactivity of black tea [9]. Recently, TFs have attracted considerable interest because of various biological activities, such as antioxidant, anti-inflammatory, anti-aging, anti-diabetic, and antimicrobial properties [8,10]. These biological activities are mainly attributed to the antioxidant abilities of TFs [11], resulting from their benzophenone skeleton and functional groups [12]. Yang et al. [13] found that TFs had a stronger scavenging efficiency than epigallocatechin gallate (EGCG) against DPPH and hydroxyl radicals. In addition, Gao et al. [14] evaluated the effect of TFs on the antioxidant capacity in cured sausage, and TFs could inhibit the oxidation of myoglobin to improve the color of cured sausage. Notedly, during the storage preservation of semi-dried large yellow croaker, TFs treatment exhibited positive effects on the stability of muscle proteins and lipids, showing excellent antibacterial activity against the genus of *Actinobacteria*, *Proteus*, and *Psychrobacter* [15]. Therefore, TFs are a potential and attractive antimicrobial agent for application in the food industry.

Food is a complex multi-scale system, including environmental factors (such as temperature, pH, and NaCl) and food matrices (such as carbohydrate, protein, and lipid), which will interfere with the antibacterial efficiency of natural preservatives. Regarding environmental factors, the antibacterial activity of thymol decreased significantly when the temperature was higher than 80 °C [16]; nisin showed a reduced antibacterial effect at acidic and basic pH compared with neutral conditions [17]; 0.2 mol/L of NaCl could significantly enhance the antibacterial activity of nisin [18]. Additionally, food matrices may have a negative influence on the antibacterial efficiency of nisin, which should be added under 60 g/L of sucrose (simulated carbohydrate-based system), 120 g/L of skimmed milk powder (simulated protein-based system), and 12 g/L of lecithin (simulated lipid-based system) in the related food products [19].

Although the antibacterial activity of TFs has been reported, their effects from different environmental factors and food matrices remain unclear. This study aims to evaluate the effects of environmental factors (temperature, pH, and NaCl) and food matrices (sucrose, skimmed milk powder, and lecithin) on the antibacterial activity of TFs against *Staphylococcus aureus* (*S. aureus*) and *Salmonella paratyphi B* (*S. paratyphi B*) through the double layer agar method. The obtained results can provide a theoretical guidance for applying TFs as a new antibacterial product in the food industry.

## 2. Materials and Methods

### 2.1. Materials

TFs (80% of purity) were provided by Jiangsu Dehe Biotechnology Co., Ltd. (Wuxi, China). Luria-Bertani (LB) broth and agar were purchased from Qingdao Haibo’ao Biotechnology Co., Ltd. (Qingdao, China). *S. aureus* (*CCMC(B) 226003*) and *S. paratyphi B* (*CMCC 50094*) in the study were kindly donated from the Food Nutrition and Microorganism Laboratory, Yangzhou University (Yangzhou, China). Skimmed milk powder, lecithin, and sucrose were supplied by Shanghai Sangong Bioengineering Co., Ltd. (Shanghai, China). Other chemicals and reagents were of analytical grade.

### 2.2. Preparation of Bacterial Culture Suspensions

*S. aureus* and *S. paratyphi B* were cultivated in LB medium for 12 h at 37 °C with 150 rpm shaking culture; then, the bacterial culture concentration was diluted to 1 × 10^7^ CFU/mL for further studies, if not otherwise stated [20,21].

### 2.3. Evaluation of Antibacterial Activity

The antibacterial activity of TFs was evaluated based on the diameter of the inhibition zone (DIZ) around Oxford cups (8.0 mm of diameter), according to the double-layer agar method described by de Azevedo et al. [22] with slight modifications. Oxford cups firstly were placed on the surface of the Agar plates; then, *S. aureus* and *S. paratyphi B* culture concentration (1 × 10^7^ CFU/mL, 250 μL) and solid media (25 mL) were thoroughly mixed into Agar plates. As the solid media solidified, the above Oxford cups were pulled out, and 150 μL of TFs solution with the concentrations of 2, 4, 6, 8, and 10 mg/mL was poured into the Oxford cup cavity. After diffusion at 4 °C for 6 h and intubation at 37 °C for 18 h, the above plates were taken out to measure DIZ with sterile water as control.

### 2.4. Effect of Environmental Factors on the Antibacterial Activity of TFs

To evaluate the antibacterial activity of TFs to pH, TFs solutions (10 mg/mL) were adjusted to pH 2–10 by adding HCl (0.4 mol/L) and NaOH (0.4 mol/L); then, their DIZ values to *S. aureus* and *S. paratyphi B* were further measured with untreated 10 mg/mL of TFs as the control [23].

TFs solutions (10 mg/mL) were treated at 25 °C (room temperature), 60 °C (lowest temperature for pasteurization), 80 °C (highest temperature for pasteurization), 100 °C (boiling temperature), and 121 °C (commercial sterilization temperature) for 30 min to evaluate the relationship between antibacterial activity of TFs and temperature. Then, their DIZ values for *S. aureus* and *S. paratyphi B* were further measured with untreated 10 mg/mL of TFs as control [24].

To explore the antibacterial activity of TFs to NaCl, TFs solutions (10 mg/mL) were prepared to 0.2–0.8 mol/L of NaCl concentrations; then, their DIZ values to *S. aureus* and *S. paratyphi B* were further measured with untreated 10 mg/mL of TFs as control [25].

### 2.5. Effect of Food Matrices on the Antibacterial Activity of TFs

Sucrose, skimmed milk powder, and lecithin were added to TFs solutions, which could be used for simulated carbohydrate-based, protein-based, and lipid-based food matrices, respectively. Then, the effects of different concentrations of sucrose (10–60 g/L), skimmed milk powder (20–120 g/L), and lecithin (2–12 g/L) on the DIZ was measured with untreated 10 mg/mL of TFs as control [19].

### 2.6. Statistical Analysis

All the tests were repeated at least three times. The data are presented as the mean ± standard deviation. Significant differences among groups were analyzed by the Duncan test at *p* < 0.05 using SPSS 20.0 software.

## 3. Results

### 3.1. Antibacterial Effects of TFs Concentrations

**Figure 1:**
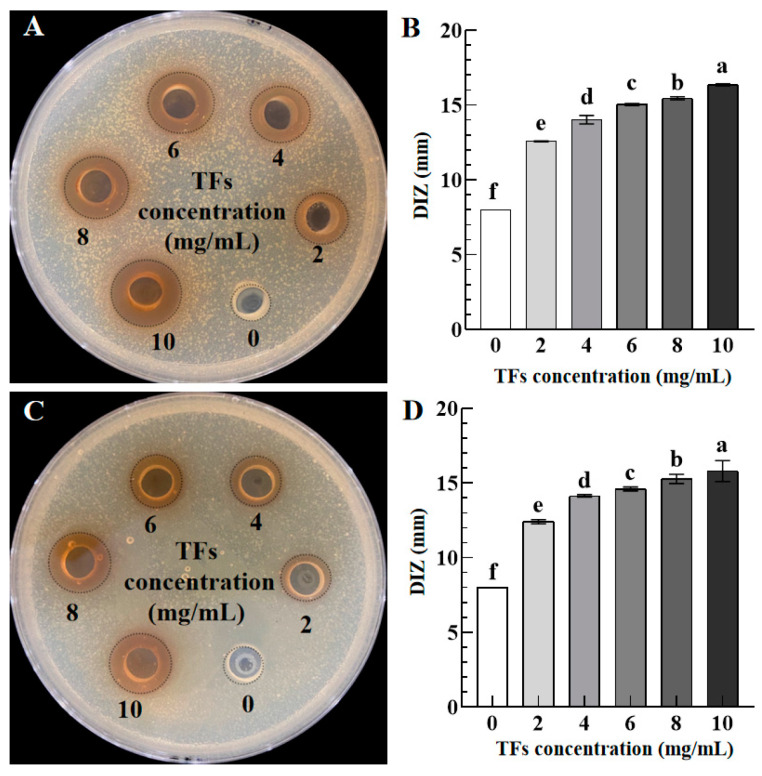
Inhibition zone appearance combined with their DIZ values of TFs against *S. aureus* (**A**,**B**), and *S. paratyphi B* (**C**,**D**) as a function of TFs concentrations (0–10 mg/mL). Different lowercase letters in (**B**,**D**) indicated significant differences among groups (*p* < 0.05).

Figure 1 exhibits the inhibition zone appearance combined with their DIZ values of TFs against *S. aureus* (A and B) and *S. paratyphi B* (C and D) as a function of TFs concentrations (0–10 mg/mL). The DIZ value without TFs was 8 mm, the same as the diameter of the Oxford cup, implying that sterile water has no antibacterial effect. As the concentration of TFs increased from 2 mg/mL to 10 mg/mL, their DIZ values increased significantly from 12.58 ± 0.09 mm to 16.36 ± 0.12 mm and 12.42 ± 0.43 mm to 15.81 ± 0.24 mm for *S. aureus* (Figure 1B) and *S. paratyphi B* (Figure 1D), respectively, showing a dose-dependent antibacterial activity of TFs. Furthermore, TFs show larger DIZ values against *S. aureus* than *S. paratyphi B* at the same concentration, consistent with the findings of EGCG, showing higher antibacterial activity on Gram-positive bacteria and low antibacterial activity on Gram-negative bacteria [26]. 

### 3.2. Effect of Environmental Factors

#### 3.2.1. Role of Temperature

**Figure 2:**
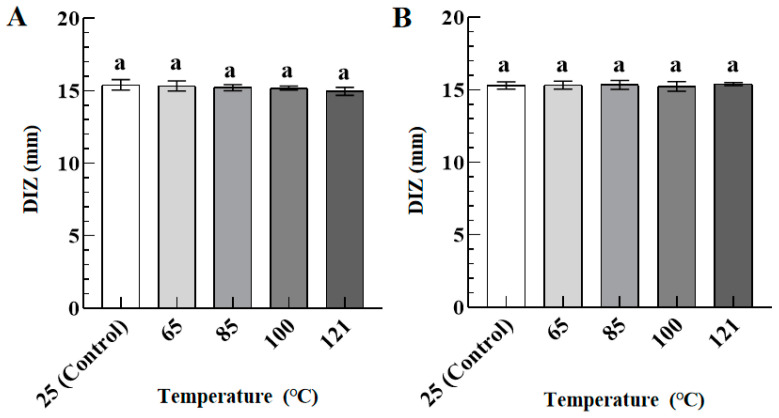
The DIZ values of TFs (10 mg/mL) against *S. aureus* (**A**) and *S. paratyphi B* (**B**) as a function of processing temperatures (25–121 °C). Different lowercase letters in (**A**,**B**) indicated significant differences among groups (*p* < 0.05).

Figure 2 shows the DIZ values of TFs against *S. aureus* (A) and *S. paratyphi B* (B) as a function of processing temperatures (25–121 °C). As temperatures increased from 25 °C to 121 °C, no significant difference was observed for the DIZ values of TFs against *S. aureus* and *S. paratyphi B*, respectively. Compared with the control group (25 °C), the high temperature at 121 °C did not show a significant effect on the antibacterial activity of TFs against *S. aureus* and *S. paratyphi B*, respectively. Therefore, TFs can be used as bio-preservatives in foods undergoing commercial sterilization. Yang et al. [19] found that the antibacterial activity in nisin was significantly reduced after high-temperature treatment, which may be attributed to the difference in molecular structure.

#### 3.2.2. Role of pH

**Figure 3:**
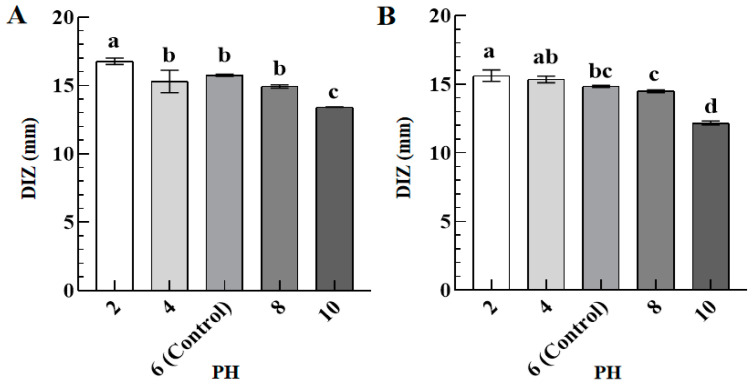
The DIZ values of TFs (10 mg/mL) against *S. aureus* (**A**) and *S. paratyphi B* (**B**) as a function of pH (2–8). Different lowercase letters in (**A**,**B**) indicated significant differences among groups (*p* < 0.05).

Figure 3 presents the DIZ values of TFs against *S. aureus* (A) and *S. paratyphi B* (B) as a function of pH (2–10). Compared with the control group (pH = 6, DIZ = 15.76 ± 0.23 mm and 14.85 ± 0.18 mm against *S. aureus* and *S. paratyphi B*), when the pH was reduced to 2, the DIZ values were increased to 16.78 ± 0.23 mm and 15.63 ± 0.42 mm for *S. aureus* and *S. paratyphi B*, implying their enhanced antibacterial activity; whereas the pH increased to 10, the DIZ values were decreased to 13.43 ± 0.08 mm and 12.18 ± 0.14 mm for *S. aureus* and *S. paratyphi B*, indicating their attenuated antibacterial activity. Therefore, low pH is beneficial for the antibacterial activity of TFs, while high pH diminishes their antibacterial activity. The result is consistent with a previous study that shows an excellent antibacterial activity of monolaurin under low pH conditions [23].

#### 3.2.3. Role of NaCl

**Figure 4:**
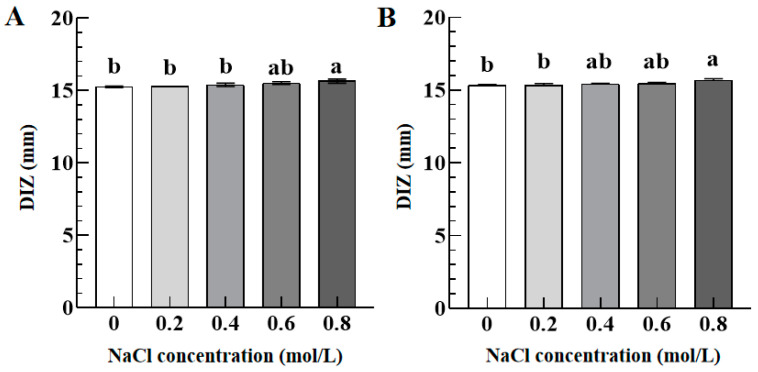
The DIZ values of TFs (10 mg/mL) against *S. aureus* (**A**) and *S. paratyphi B* (**B**) as a function of NaCl concentrations (0–0.8 mol/L). Different lowercase letters in (**A**,**B**) indicated significant differences among groups (*p* < 0.05).

Figure 4 illustrates the DIZ values of TFs against *S. aureus* (A) and *S. paratyphi B* (B) as a function of NaCl concentrations (0–0.8 mol/L). There was no significant difference in their DIZ values against *S. aureus* and *S. paratyphi B* when the NaCl concentration increased from 0 to 0.6 mol/L. The DIZ values increased slightly from 15.44 ± 0.10 mm and 15.65 ± 0.05 mm to 15.50 ± 0.11 mm and 15.67 ± 0.09 mm against *S. aureus* and *S. paratyphi B* when the NaCl concentration increased from 0.6 mol/L to 0.8 mol/L. Compared to the control group (NaCl concentration 0 g/L), TFs with 0.8 mol/L of NaCl concentration showed a significantly enhanced antibacterial activity against *S. aureus* and *S. paratyphi B*. Therefore, the antibacterial activity of the TFs was enhanced when the NaCl concentration reached above 0.8 mol/L. These findings are consistent with a previous study [27], which showed that combining NaCl with essential plant oils (carvacrol and thymol) significantly increased the antibacterial activity against *E. coli O157:H7*, *Listeria monocytogenes*, and *S. aureus*. The result is caused by the synergistic effect of antibacterial activity resulting from multiple stress factors. Therefore, TFs and NaCl show a synergistic antibacterial activity against *S. aureus* and *S. paratyphi B*.

### 3.3. Effect of Food Matrices

#### 3.3.1. Role of Sucrose

**Figure 5:**
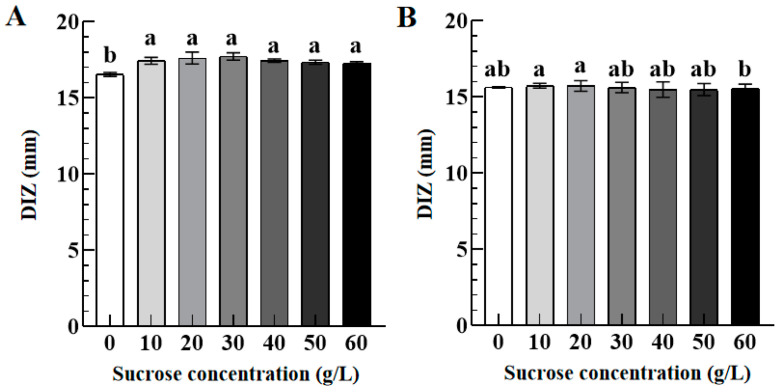
The DIZ values of TFs (10 mg/mL) against *S. aureus* (**A**) and *S. paratyphi B* (**B**) as a function of sucrose concentrations (0–60 g/L). Different lowercase letters in (**A**,**B**) indicated significant differences among groups (*p* < 0.05).

Figure 5 shows the DIZ values of TFs against *S. aureus* (A) and *S. paratyphi B* (B) as a function of sucrose concentration (0–60 g/L). As sucrose concentration increased from 0 to 10 g/L, the DIZ value of TFs against S. aureus increased significantly from 16.55 ± 0.19 mm to 17.44 ± 0.15 mm, and no significant difference was observed for their DIZ values with sucrose concentration further increasing to 60 g/L. Additionally, there was no significant change in the DIZ value of TFs against *S. paratyphi B* with sucrose concentration ranging from 0 to 60 g/L. Compared with the control group (sucrose concentration 0 g/L), TFs with 60 g/L of sucrose concentration exhibited an enhanced antibacterial activity against *S. aureus* and a neutral antibacterial activity against *S. paratyphi B*. The result implies that sucrose addition shows a stronger influence on the antibacterial activity of TFs against *S. aureus* than *S. paratyphi B*, which may be attributed to various species displaying different susceptibilities to the same antimicrobial agent, resulting in a strain-specific effect of sucrose on antibacterial activity [28].

#### 3.3.2. Role of Skimmed Milk Powder

**Figure 6:**
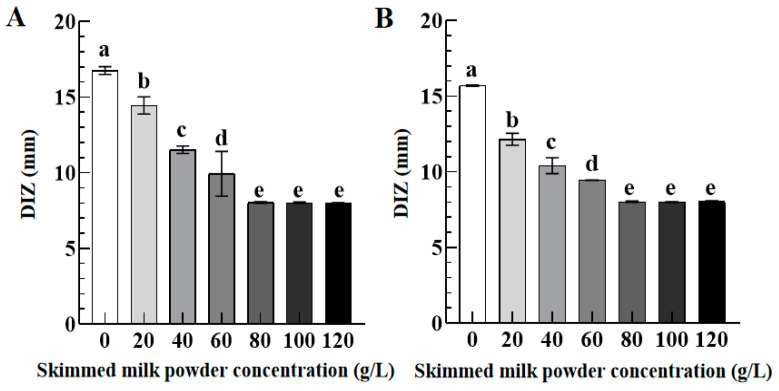
The DIZ values of TFs (10 mg/mL) against *S. aureus* (**A**) and *S. paratyphi B* (**B**) as a function of skimmed milk powder concentrations (0–120 g/L). Different lowercase letters in (**A**,**B**) indicated significant differences among groups (*p* < 0.05).

Figure 6 presents the DIZ values of TFs against *S. aureus* (A) and *S. paratyphi B* (B) as a function of skimmed milk powder concentrations (0–120 g/L). As skimmed milk powder concentration increased from 0 to 80 g/L, the DIZ values of TFs against *S. aureus* and *S. paratyphi B* decreased significantly from 16.81 ± 0.68 mm and 15.76 ± 0.08 mm to 8.00 ± 0.08 mm and 8.00 ± 0.12 mm, respectively. However, when the concentration of skimmed milk powder further increased to 120 g/L, there was no significant change in the DIZ values of TFs against both *S. aureus* and *S. paratyphi B*. The above results indicated that TFs with 20–120 g/L of skimmed milk powder concentration showed an attenuated antibacterial activity against *S. aureus* and *S. paratyphi B* as compared to the control group (skimmed milk powder concentration 0 g/L). These results suggest that skimmed milk powder has a negative impact on the antibacterial activity of TFs because protein is an essential nutrient for microbial growth. The antibacterial activity of resveratrol in milk is similar [29].

#### 3.3.3. Role of Lecithin

**Figure 7:**
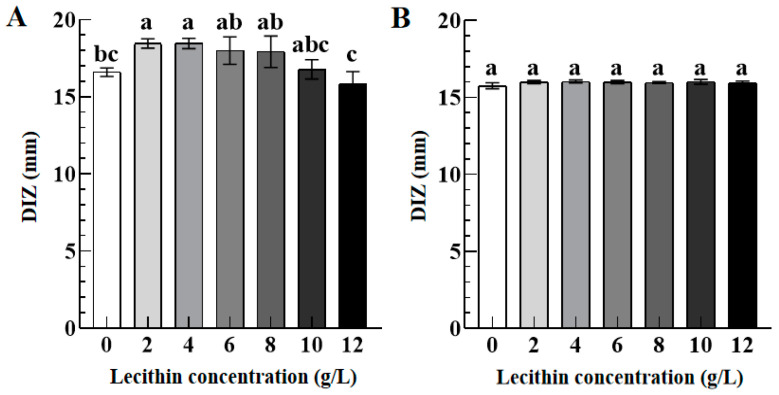
The DIZ values of TFs (10 mg/mL) against *S. aureus* (**A**) and *S. paratyphi B* (**B**) as a function of lecithin concentrations (0–12 g/L). Different lowercase letters in (**A**,**B**) indicated significant differences among groups (*p* < 0.05).

Figure 7 exhibits the DIZ values of TFs against *S. aureus* (A) and *S. paratyphi B* (B) as a function of lecithin concentrations (0–12 g/L). Compared with the control group (lecithin concentration 0 g/L), the DIZ value of TFs against *S. aureus* increased significantly from 16.61 ± 0.28 mm to 18.46 ± 0.33 mm when the lecithin concentration increased from 0 to 2 g/L, implying their enhanced antibacterial activity of TFs against *S. aureus*. However, as the lecithin concentration further increased to 10 g/L and 12 g/L, their DIZ values of TFs against *S. aureus* decreased significantly to 16.78 ± 0.89 mm and 15.86 ± 1.02 mm with no significant difference as compared to the control group. In addition, there is no significant difference in their DIZ values against *S. paratyphi B* when the lecithin concentration increased from 0 to 12 g/L. Our findings are consistent with a previous study that quercetin-enriched lecithin exhibits an antibacterial activity compared with lecithin or quercetin [30].

## 4. Discussion

**Figure 8:**
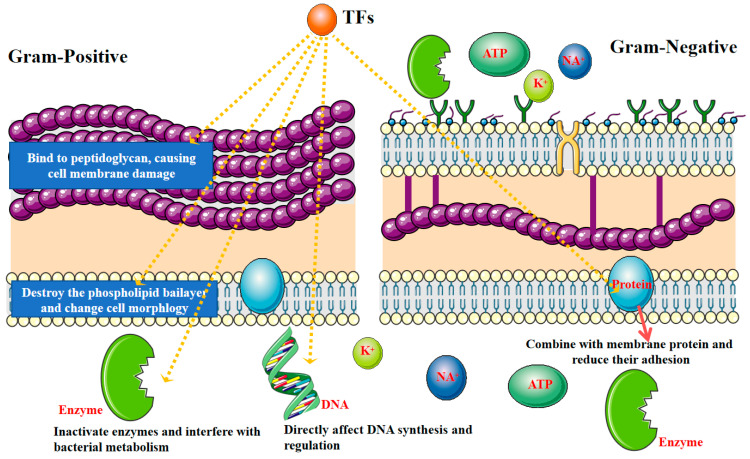
Possible antibacterial mechanism of TFs against Gram-positive and Gram-negative bacteria [32,33,34].

In this study, TFs exhibited excellent antibacterial activity against *S. aureus* (Gram-positive bacteria) and *S. paratyphi B* (Gram-negative bacteria). Due to their polycyclic structures and phenolic hydroxyl groups, TFs have a high affinity for biomacromolecules such as lipids, proteins, carbohydrates, and nucleic acids. This high affinity enables TFs to react with the bacterial cell membrane, resulting in an unstable membrane structure, decreased membrane fluidity, and destroyed membrane integrity [31], as shown in Figure 8.

TFs showed a larger DIZ value against *S. aureus* than *S. paratyphi B* at the same concentration, exhibiting a stronger antibacterial activity against *S. aureus*. The result may be related to their differences in the structures and compositions of the cell wall [35]. Similar results were observed for EGCG [36] and catechin [37], wherein they could cause cell membrane damage easier in Gram-positive bacteria with a thin peptidoglycan layer. Furthermore, Gram-negative bacteria had an additional outer membrane mainly composed of lipopolysaccharides overlaying the thin peptidoglycan layer, which may be a fundamental reason for the weak inhibitory effect against Gram-negative bacteria [38]. These results should be responsible for the role of environmental factors (processing temperature, pH, and NaCl) and food matrices (sucrose, skimmed milk powder, and lecithin) in the antibacterial effects of TFs against *S. aureus* and *S. paratyphi B*. 

As for the effect of processing temperature (25–121 °C) on the antibacterial activity of TFs, there was no significant change, even at high temperatures commonly used in commercial sterilization processes. Bacteriocin remained active after autoclaving at 121°C for 15 min [39]. Additionally, Su et al. [40] reported that the mixture of catechins and TFs may suffer severe degradation from 100 °C for 3 h. However, no significant degradation of TFs could occur at the processing temperatures of 100 °C and 121 °C for 30 min in this study, resulting in no change for the antibacterial activity of TFs. The results may be attributed to their detailed compositions and limited processing time. The above results indicate that TFs possess an excellent antibacterial activity to high processing temperatures, which is a crucial factor in preserving heat-processed food products with good antibacterial properties.

**Figure 9:**
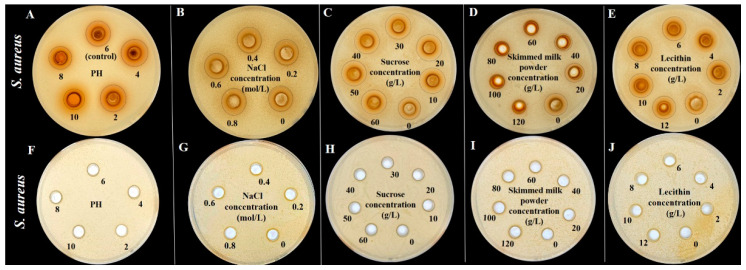
Effect of pH (2–8), NaCl concentrations (0–0.8 mol/L), sucrose concentrations (0–60 g/L), skimmed milk powder concentrations (0–120 g/L), and lecithin concentrations (0–12 g/L) with (**A**–**E**) and without (**F**–**J**) TFs on the inhibition zone appearance from *S. aureus*.

**Figure 10:**
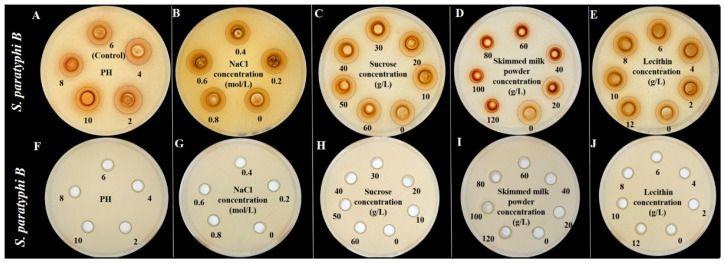
Effect of pH (2–8), NaCl concentrations (0–0.8 mol/L), sucrose concentrations (0–60 g/L), skimmed milk powder concentrations (0–120 g/L), and lecithin concentrations (0–12 g/L) with (**A**–**E**) and without (**F**–**J**) TFs on the inhibition zone appearance from *S. paratyphi B*.

In addition, this study investigated the effect of environmental factors (pH and NaCl) and food matrices (sucrose, skimmed milk powder, and lecithin) on the antibacterial activity against *S. aureus* and *S. paratyphi B*, as shown in Figure 9 and Figure 10, respectively. There is no significant difference for the inhibition zone appearance with pH values (2–8), NaCl concentrations (0–0.8 mol/L), sucrose concentrations (0–60 g/L), skimmed milk powder concentrations (0–120 g/L), and lecithin concentrations (0–12 g/L), implying their insufficient antimicrobial activity. Interestingly, a remarkable antibacterial effect with an apparent inhibition zone was observed for the above conditions with TFs addition, which should be responsible for antimicrobial activity. These findings are consistent with previous studies that phenolic-rich plant extracts or pure plant phenolics could inhibit the growth of microorganisms in the special food matrix (such as chicken soup and pasteurized milk) [41,42,43]. 

Considering the effect of pH (2–10) on the antibacterial activity of TFs, *S. aureus* and *S. paratyphi B* both exhibited an increased sensitivity at low pH values of 2–6, which may be due to their damaged cell membrane (e.g., membrane permeability or lipid peroxidation) in an acidic environment [44]. This phenomenon is consistent with the findings of Rda et al. [45] and Buldain et al. [46]. They found that Thurincin H and essential oils were more effective in inhibiting the growth of *Listeria innocua* and *Escherichia coli* at pH 5–6.5, respectively. Furthermore, Lee et al. [47] found that TFs were stable at pH 6.5 and degraded seriously at pH 9, resulting in the decreased antibacterial activity. Therefore, TFs are more suitable for food processing and preservation in an acidic environment than an alkaline environment, which is conducive to the efficient performance of their antibacterial activity.

Considering the effect of NaCl concentration (0.2–0.8 mol/L) on the antibacterial activity of TFs, TFs and NaCl exhibited a synergistic antibacterial activity against *S. aureus* and *S. paratyphi B*, especially at a concentration of 0.8 mol/L NaCl. Xu et al. [48] observed that the biofilm formation of *S. aureus* was seriously suppressed with the increasing NaCl concentration (0–2%). This finding was consistent with the previous studies where NaCl could enhance the efficiency of *Ruta chalepensis* essential oils in killing microorganisms under certain conditions [49]. In addition, Wen et al. [50] applied tea polyphenols to preserve pork sausages for high-salt foods, and found that tea polyphenols could enable their excellent quality and sensory characteristics, and prolonged the shelf-life from 36 d to 42 d. Therefore, high concentrations (>0.8 mol/L) of NaCl combined with TFs are encouraged for food processing and preservation.

Regarding the effect of sucrose concentration (10–60 g/L) on the antibacterial activity of TFs, sucrose addition significantly enhanced the antibacterial activity of TFs against *S. aureus*, but no significant change for *S. paratyphi B*, which may be attributed to the species-specific effects resulting from the varied susceptibilities of different species to the same antibacterial agent [51]. The increase in osmotic pressure caused by high sucrose concentration may lead to an imbalance in microbial cells, thus, increasing their susceptibility to TFs [52]. Yang et el. [19] reported that the antibacterial activity of nisin against *Listeria monocytogenes* was significantly reduced when the sucrose concentration increased to 60 g/L, which may be attributed to the molecular structure of the antibacterial agent and its interaction with sucrose [28]. The above results suggest that TFs are suitable for preservation and freshness maintenance in carbohydrate-based systems, such as sucrose concentration < 60 g/L.

As for the effect of skimmed milk powder concentration (20–120 g/L) on the antibacterial activity of TFs, their antibacterial activity against *S. aureus* and *S. paratyphi B* was significantly decreased when the skimmed milk powder concentration increased from 20 g/L to 80 g/L, which could be attributed to the interaction between polyphenols and proteins resulting in the formation of polyphenol–protein complexes, thereby limiting the action of active polyphenolic compounds against microbial cells [53]. Smith et al. [54] further reported that protein could weaken the antibacterial activity of active ingredients by promoting the growth of bacteria. Therefore, the concentration of skimmed milk powder should be controlled within 80 g/L to facilitate the antibacterial activity of TFs in a protein-based system.

Considering the effect of lecithin concentration (2–12 g/L) on the antibacterial activity of TFs, their antibacterial activity against *S. aureus* exhibited a significant increasing trend, but no significant change for *S. paratyphi B*. The results may be because the combination of lecithin with Ca^2+^ and Mg^2+^ destabilizes the lipopolysaccharide membrane [55]. Furthermore, the antibacterial activity of the quercetin-enriched lecithin formulation was better than that of individual quercetin, showing high antibacterial activity against Gram-positive [30]. Additionally, adding tea polyphenols to edible fats, oils, and fat-containing products could effectively inhibit lipid oxidation, and the growth and reproduction of microorganisms, ultimately extending the shelf-life of such products [56,57]. Therefore, this study provides a theoretical reference for applying TFs in the preservation and freshness maintenance of a lipid-based system (lecithin concentration < 12 g/L).

## 5. Conclusions

In this study, TFs had better antibacterial activity against *S. aureus* than *S. paratyphi B*. TFs also exhibited a dose-dependent antibacterial activity, and showed an ideal antibacterial activity with high processing temperatures and low pH conditions. Additionally, 0.8 mol/L NaCl had a synergistic effect on the antibacterial activity of TFs. In different food matrices, TFs maintained good antibacterial activity in the concentration range of sucrose (<60 g/L), skimmed milk powder (<80 g/L), and lecithin (<12 g/L). This study provides valuable theoretical support for the application of TFs in the food industry, which is beneficial for developing food-grade antimicrobial agents in food preservation.

## Data Availability

The data used to support the findings of this study can be made available by the corresponding author upon request.

## Abbreviations

DIZ: diameter of inhibition zone; DPPH: 2,2-diphenyl-1-picrylhydrazyl; EGCG: epigallocatechin gallate; LB: Luria-Bertani; TFs: theaflavins.
